# Bluetongue Disease Risk Assessment Based on Observed and Projected *Culicoides obsoletus* spp. Vector Densities

**DOI:** 10.1371/journal.pone.0060330

**Published:** 2013-04-01

**Authors:** Katharina Brugger, Franz Rubel

**Affiliations:** Institute for Veterinary Public Health, University of Veterinary Medicine Vienna, Vienna, Austria; Institute for Animal Health, United Kingdom

## Abstract

Bluetongue is an arboviral disease of ruminants causing significant economic losses. Our risk assessment is based on the epidemiological key parameter, the basic reproduction number. It is defined as the number of secondary cases caused by one primary case in a fully susceptible host population, in which values greater than one indicate the possibility, i.e., the risk, for a major disease outbreak. In the course of the Bluetongue virus serotype 8 (BTV-8) outbreak in Europe in 2006 we developed such a risk assessment for the University of Veterinary Medicine Vienna, Austria. Basic reproduction numbers were calculated using a well-known formula for vector-borne diseases considering the population densities of hosts (cattle and small ruminants) and vectors (biting midges of the *Culicoides obsoletus* spp.) as well as temperature dependent rates. The latter comprise the biting and mortality rate of midges as well as the reciprocal of the extrinsic incubation period. Most important, but generally unknown, is the spatio-temporal distribution of the vector density. Therefore, we established a continuously operating daily monitoring to quantify the seasonal cycle of the vector population by a statistical model. We used cross-correlation maps and Poisson regression to describe vector densities by environmental temperature and precipitation. Our results comprise time series of observed and simulated *Culicoides obsoletus* spp. counts as well as basic reproduction numbers for the period 2009–2011. For a spatio-temporal risk assessment we projected our results from the location of Vienna to the entire region of Austria. We compiled both daily maps of vector densities and the basic reproduction numbers, respectively. Basic reproduction numbers above one were generally found between June and August except in the mountainous regions of the Alps. The highest values coincide with the locations of confirmed BTV cases.

## Introduction

Veterinary authorities are generally interested in a spatio-temporal risk assessment to establish efficient protection and control measures when facing diseases with high economic impact such as caused by the Bluetongue virus (BTV). Such risk assessments are frequently based on one common epidemiological parameter, the basic reproduction number 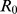. It describes the number of secondary cases caused by a primary case in a completely susceptible population at the beginning of an epidemic. Thus, 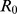 may be interpreted as a threshold parameter: a major disease outbreak may only occur for R
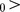
1, while for R
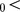
1 the disease will fade out [1].

**Figure 1:**
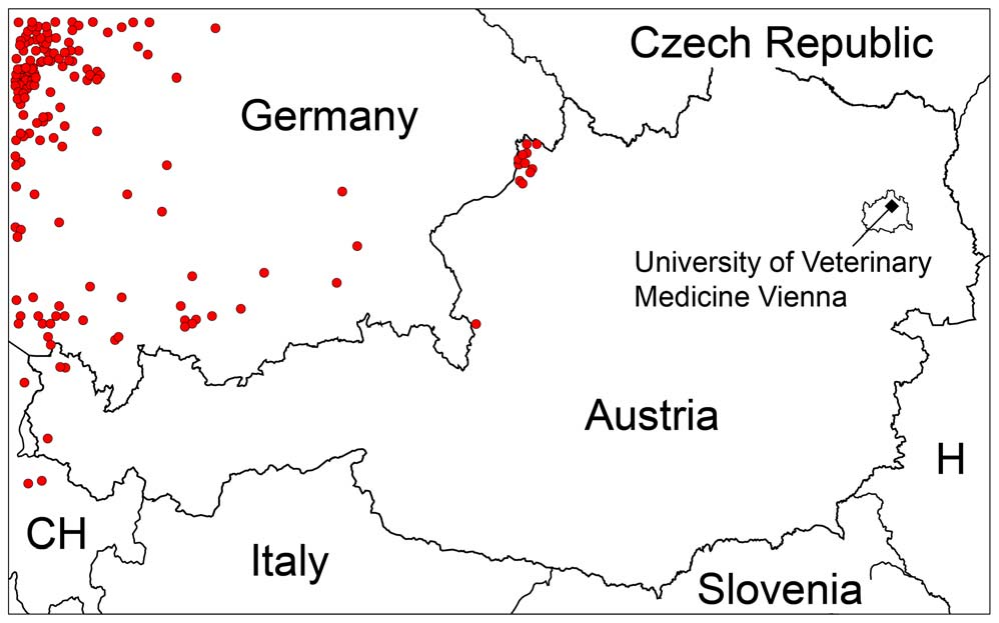
Reported locations of BTV-8 cases between 2006 and 2009 in Germany [8], Austria [43] and Switzerland [44].

The motivation for this study was the Bluetongue virus serotype 8 (BTV-8) epidemics in Europe, which emerged in 2006 at the border triangle of Belgium, Germany and the Netherlands [2], [3]. Within a few years this arboviral disease rapidly spread across North-western and Central Europe [4], [5] and caused substantial losses in agriculture amounting to millions Euro [6]. Beside movement restrictions and surveillance, the main veterinary measure was to vaccinate the susceptible populations beginning in 2008 [7]. In Austria, only a few cattle near the border to Germany were confirmed to be BTV-8 seropositive. Due to the German vaccination campaigns achieving a coverage of 78% [8] and preventive vaccination in Austria the eastwards spread of the Bluetongue disease could be stopped at the German-Austrian border (Figure 1).

In the course of the European BTV-8 outbreak several methods for the calculation of 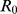 have been proposed. Some of these methods are based on data from surveillance and monitoring programmes. For example, [9] estimated 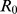 between herds for the Netherlands. Furthermore, a theoretical study on 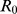 using a deterministic epidemic model was introduced by [10] to investigate the seasonal spread of BTV over several years and to evaluate the effectiveness of vaccination strategies. Most approaches presented so far considered temperature dependent vector parameters as well as host and vector densities. While [11] presented a general concept on the calculation of 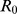 for which they discussed the influence of temperature on the transmission cycle, [12] and [13] generated spatial 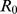 maps for the Netherlands and Switzerland, respectively. Furthermore, the uncertainty and sensitivity of such temperature-dependent models have been analysed and temperature turned out to be the most dominant factor for determining the magnitude of 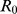 [14]. [15] estimated the possible impact of past and future climates on the basic reproduction number 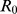 on an European scale. For their study they used input data from climate model runs applied to different emission scenarios of the Intergovernmental Panel on Climate Change (IPCC). The authors have used a similar approach for modelling another mosquito-borne disease outbreak, the Usutu virus epidemics in Vienna [16]. Here we present a predictive risk assessment method based on the basic reproduction number 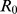 after [12] applied to a potential Bluetongue outbreak in Austria. Results comprise regions at risk on a spatio-temporal scale representative for a resolution of 10 km and 1 day.

In general, BTV circulates in a natural transmission cycle between vectors (*Culicoides* biting midges) and hosts (ruminants: mainly cattle, sheep, and goats). For risk assessment using the concept of 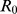 the knowledge of both the vector and the host densities are of fundamental importance. While the population densities of farm animals are well documented in national veterinary databases or available worldwide on a regular grid [17], the vector density is generally not well known or rather unknown. Therefore, a central part of our study is concerned with the estimation of the vector population. We established a continuously operating daily *Culicoides spp.* monitoring at the University of Veterinary Medicine Vienna, Austria, to quantify the seasonal abundance for at least three years. This approach is in contrast to former - mainly weekly - monitoring programs, such as the large-scale entomological surveillance program throughout the European Union to investigate the occurrence and geographical distribution of *Culicoides spp.* ([18]; results were published e.g. by [19], [20]).

## Methods

### Vector monitoring

For the *Culicoides spp.* monitoring a black light suction trap of Onderstepoort type, as described by [21], was placed next to an automatic weather station at the university campus (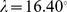
E geographical longitude and 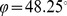
N geographical latitude). The location in Vienna is characterised by a temperate, fully humid climate with warm summers, corresponding to Cfb climate following the Köppen-Geiger climate classification [22]. This standardized trap is commonly used in monitoring and surveillance programs, because it is very efficient compared to other suction light traps [23]. Following the guideline of [24], the trap was hung at a height of 1.5 m above the ground. The distance to the stables and paddocks with cattle, sheep, and horses was less than 20 m. A collection bottle rotator (model 1512, John W. Hock Company, FL, USA) with eight beakers was used to segregate collections at 24 h intervals. For species evaluation the catches were separated first in *Culicoides spp.* and other insects (bycatches) under a stereomicroscope. Afterwards the *Culicoides* species complexes were determined by the characteristic pattern and coloration of the wings according to [24]–[26].

During the monitoring period 2009–2011 a total of 18,952 *Culicoides spp.* were captured, in which the predominantly trapped complex was *C. obsoletus* (85.7%). The *C. obsoletus* complex comprises *C. obsoletus*, *C. scoticus*, *C. dewulfi*, and *C. chiopterus*. In addition we identified midges of the *C. pulicaris* complex (9.8%), amongst others (4.5%). In this study we used only the most frequent vectors, i.e. those of the *C. obsoletus* complex.

### Vector modelling

The aim of our vector modelling is to statistically describe the midge catches by operationally available meteorological quantities. Assuming that the relationship between meteorological quantities and *C. obsoletus* abundance is location-independent (but not independent on the host density), the model will be used to estimate the spatial distribution of *C. obsoletus*. Additionally, the *C. obsoletus* abundance may be projected into the future by using meteorological output parameters from numerical weather prediction models. The development of the statistical model was carried out in two steps. In the first step, we performed a cross correlation map (CCM) analysis to investigate which of the meteorological quantities correlates best with the observed *C. obsoletus* counts. In the second step, we used these meteorological quantities in a Poisson regression model. This procedure was done first for Vienna to demonstrate the performance of the statistical model by comparing it's output with observations and subsequently applied for the entire region of Austria.

**Figure 2:**
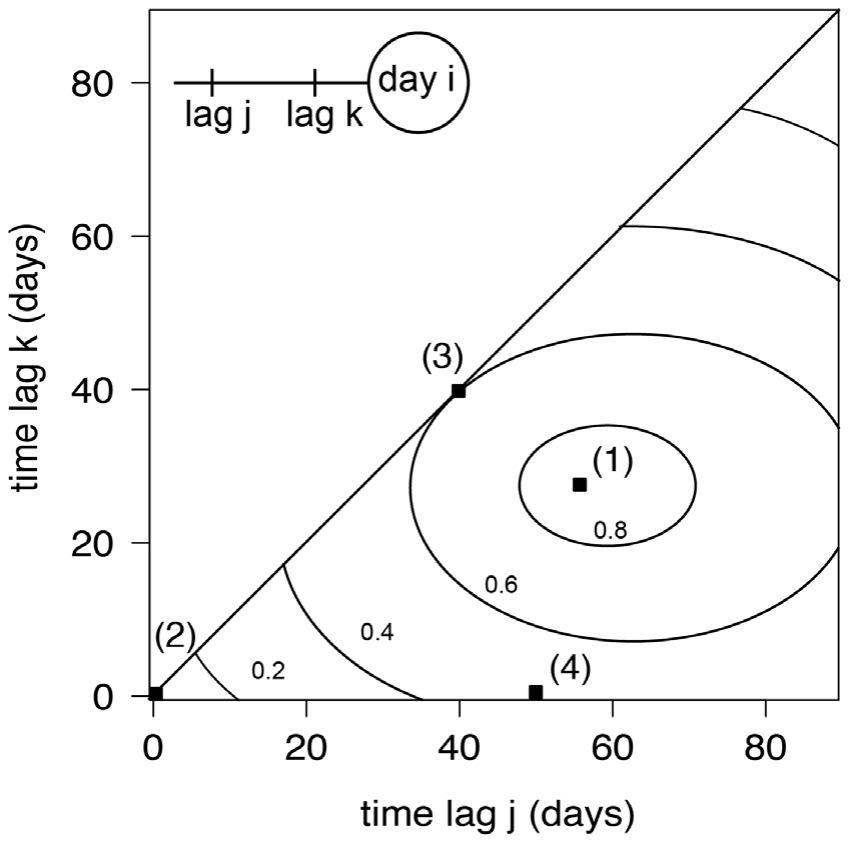
Schematic illustration of a cross correlation map (CCM). (1) Maximum correlation of 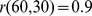 for a dependent vs. an independent quantity. The latter averaged over the period of preceding days j = 60 to k = 30. (2) Commonly used correlation coefficient (without time lags, j = k = 0) of 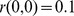. (3) Cross-correlation (with one time lag, j = k = 40) of 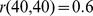, which is equal to a cross correlation with a single time lag. (4) Cross correlation of r(50,0), the independent quantity was averaged over the preceding 50 days.

The CCM analysis is an extension of the ordinary cross-correlation by introducing a second time lag. This second time lag is used to average or accumulate meteorological quantities over a period beginning at time lag 1 and ending at time lag 2. Correlation coefficients are then calculated for these averaged quantities, in which the CCMs depict the averaging period resulting in the highest correlation coefficient (Figure 2). CCMs were adopted for example by [27] to investigate the relationship between *Ochlerotatus sollicitans* abundance and environmental parameters. Further applications comprise studies on *Aedes sollicitans* [28] as well as *Aedes sollicitans* and *Culex salinarius* [29].

Using a more mathematical notation, the statistical analysis reads as follows: For 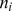 representing a time series of the observed population of midges and 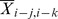 a meteorological quantity with time index 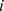, the CCM considers 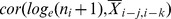 for each time lag 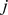 and 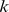, at which 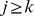. Generally, the population data were normalized using a 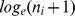-transformation and 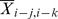 is averaged over the time interval ranging from 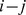 to 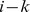. The correlations for all possible time intervals are displayed in a CCM with the time lags 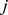 and 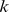 range along the two axes. Note, that for the special case of 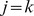 (45-line in a CCM), the correlation coefficients are equal to those of a cross-correlogram. We developed a source code for the calculation of CCMs using the R statistical computing environment [30].

Based on the correlations found by CCMs, a Poisson regression was applied to describe daily vector abundance by meteorological quantities. Again, the analysis was performed using the R statistical computing environment.

### Risk assessment

For the different tasks we used vector densities, estimated from both observed and simulated counts of midges, to assess the risk of a potential BTV-8 outbreak in cattle and small ruminants. Therefore, we applied the basic reproduction number 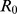, which may be interpreted as a threshold value for the possibility of a major outbreak (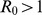). An analytical solution for 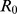 is generally derived from epidemic differential equation models. Here the formula used in recent studies by [12], [15] and [31] is applied, which may be traced back to the historical developments by Ronald Ross and George Macdonald [32].
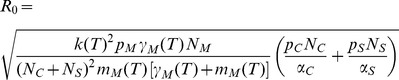
(1)



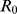
 equations given by [12], [15] and [31] are formally equivalent but differ in the complexity of parameter formulation. The most comprehensive formulation by [31] provides a gamma distribution for the extrinsic incubation period, which reduces to equation (1) by setting the scale parameters for the gamma distributions to one. Model parameters are probabilities for an infection of cattle 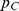, small ruminants 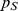 and midges 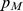, the removal rate (recovery or death rate) for cattle 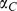 and small ruminants 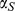, as well as some functions describing the temperature dependent behaviour of midges. The latter comprise the biting rate of midges 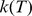, the virus reproduction rate in midges 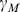
(T), i.e. the reciprocal of the extrinsic incubation period, and the mortality rate of midges 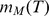. All constant parameters as well as temperature dependent parameter functions were taken according to [12] and are summarized in Table 1. Note that the temperature dependent parameter functions are not well defined for low temperatures. Therefore, we calculated 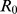 only for threshold values of 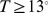 C. Note further that several other functions were proposed in the literature. A sensitivity study performed by [33] using alternative functions for midge mortality, extrinsic incubation period and biting rate results in similar 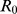 values (see Text S1 for details). Additionally, the basic reproduction number depends on the host and vector densities. We distinguish between cattle 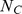, small ruminants 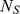 and midges 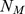.

**Table 1:** Parameters and parameter functions as applied to calculate the basic reproduction numbers 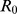.

Parameter	Symbol	Value/Function	Reference
transmission probability to midges	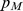	1.00	[12]
transmission probability to hosts	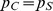	0.05	[12]
removal rate of cattle	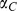	0.055	[31]
removal rate of small ruminants	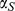	0.125	[12]
biting rate of midges	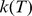	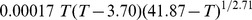	[45]
virus reproduction rate in midges	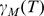	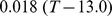	[46]
mortality rate of midges	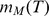	0.0089 exp 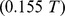	[47]

## Results

### Simulated vector population

**Figure 3:**
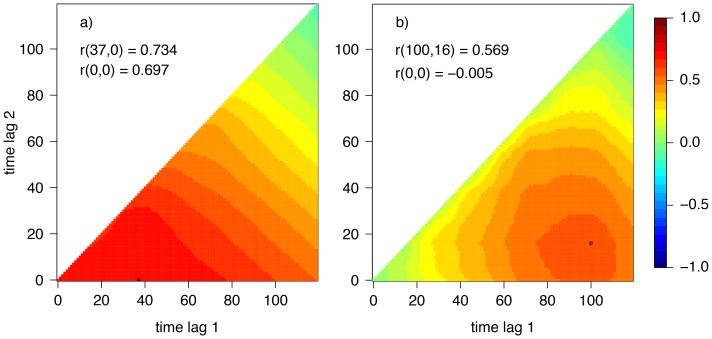
Cross correlation maps (CCMs) for daily time series in Vienna, Austria: Midges vs. temperature (a) and midges vs. precipitation (b). Note that midge counts and precipitation values were normalized by log-transformation. Period: Jan. 2009 to Dec. 2011.

In a first step we investigated the correlations between time series of midges of the *Culicoides obsoletus* complex and environmental quantities measured by the automatic weather station beside the light trap. The latter comprise amongst others air temperature, precipitation, wind speed and relative humidity. CCMs as discussed above were compiled. In accordance with the life cycle of midges we considered time frames of 120 days (4 months) preceding the catches. From all investigated quantities only temperature and precipitation were found to correlate quite well with the daily midge counts. Note that daily precipitation has a skewed frequency distribution and was normalized by log-transformation before CCMs were calculated (Figure 3).

**Figure 4:**
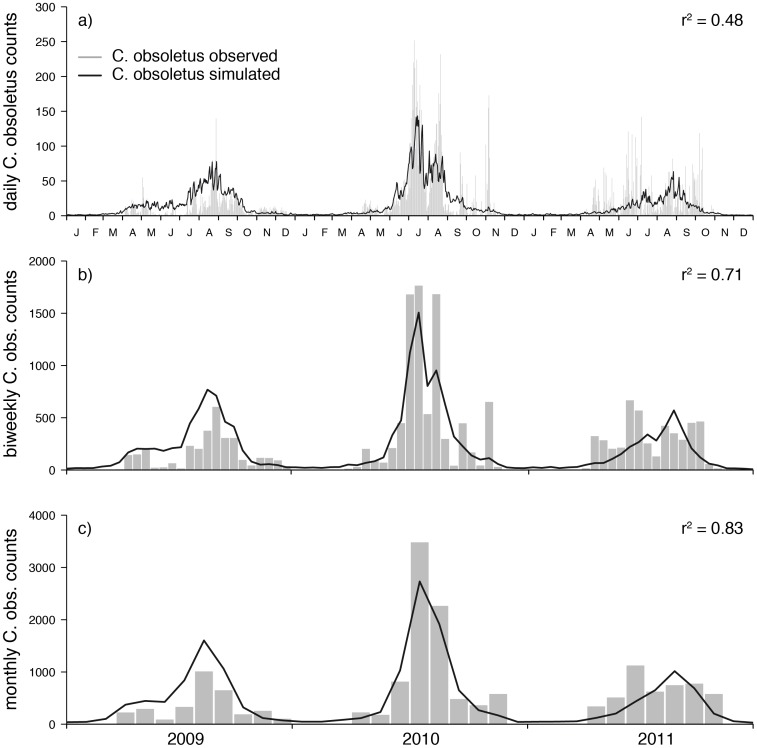
Daily (a), biweekly (b) and monthly (c) *Culicoides obsoletus* complex counts observed with the black-light trap (bars) vs. predicted by the Poisson regression model (lines). Period: January 2009 to December 2011; Vienna, Austria.

We found a maximum correlation for midges vs. temperature averaged over the preceding 37 days of 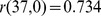 (Figure 3 left). This correlation is slightly higher than the corresponding day-to-day correlation of r(0,0) = 0.697. Between midges and precipitation we found no day-to-day correlation, but for the precipitation averaged over the period between the preceding days 100 to 16 a correlation of r(100,16) = 0.569 was calculated (Figure 3 right). Inserting these three positive correlations into the Poisson regression model leads to
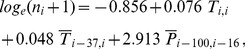
(2)with the daily temperature 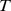, the mean temperature 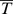, and the mean logarithmic precipitation 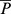 (contribution of all coefficients significant with 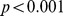). As a result, observed vs. predicted daily *Culicoides* counts for Vienna are depicted in Figure 4. According to that, temperature and precipitation explain about 48.1% of the daily variance of the *C. obsoletus* counts. We plotted also bi-weekly and monthly time series, although it is clear that the explained variances are higher for accumulated data, because they are usually appropriate for many epidemiological applications. For monthly data, for example, the explained variance increases up to 83.3%.

### Dynamic Bluetongue risk assessment for Vienna

We calculated the daily reproduction number 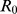 for Vienna by applying Eq. 1, in which vectors and hosts may be alternatively inserted as absolute numbers or densities. Assuming our university campus covers an area of 1 km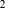, values given in absolute numbers or densities are equal. We used an average host density of cattle and small ruminants of 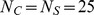 animals/km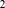. The true vector density 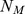, however, is unknown. In every case it is much higher than the number of midges caught at the location of the light trap. In the absence of any other estimate we applied the reasonable assumption proposed by [12]. Accordingly, the trapped midges were assumed to reflect 1% of the local vector population, i.e. our catches were multiplied by a factor of 100 before entering Eq. 1 (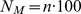). Additionally, the temperature dependent functions given in Table 1 are defined for temperatures 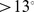 C, hence 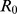 is calculated for only 46.3% of the days (mainly between April and October).

**Figure 5:**
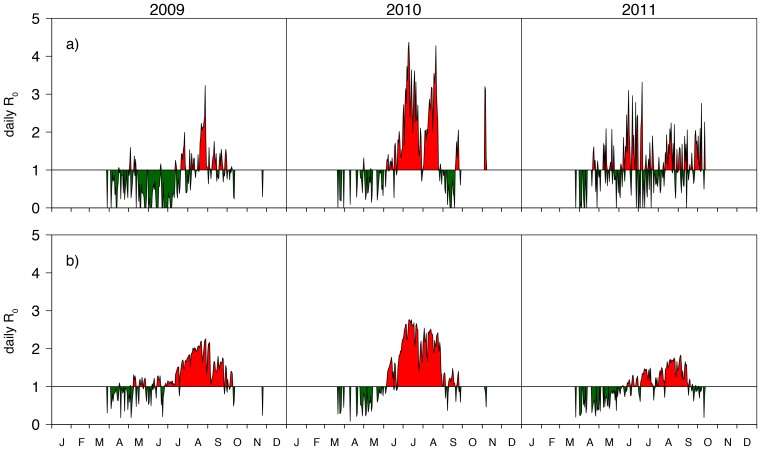
Daily basic reproduction number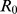 for Vienna based on observed (a) and simulated (b) *Culicoides obsoletus* time series. Red and green bars indicate 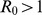 and 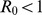, respectively.

Figure 5 depicts the time series of the daily reproduction number 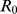 for Vienna. While in Figure 5a the observed numbers of midges were used, Figure 5b depicts 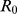 values based on the simulated numbers of midges. Taking the first as a gold standard we evaluated the influence of simulated midges 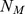 on the calculation of 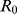 by using the two verification measures sensitivity and specificity. We calculated a sensitivity of 0.81 (probability that days with 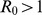 were correctly realized using 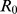 estimates based on simulated 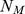) and a specificity of 0.53 (probability that days with 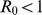 were correctly realized).

### Spatio-temporal Bluetongue risk assessment for the entire region of Austria

Assuming that 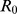 calculations in Vienna based on simulated 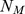 values satisfy our requirements concerning the accuracy, we applied the 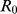 estimation method to the entire region of Austria. Therefore we defined a grid with 10 km spacing, equidistant in geographical longitude (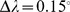) and latitude (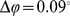). Gridded data of temperature and precipitation were taken from the Austrian meso-scale numerical weather prediction model ALADIN [34]. Only data for the period 2010–2011 were used, because data for earlier years were not available. The advantage of ALADIN data over observations (in situ or satellite data) is that they are available on a higher temporal resolution, will be available in near future also on a much higher spatial resolution and gives us the opportunity to project 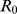 values to the future. It should be mentioned that we found a few extraordinary high precipitation values within the ALADIN data leading to unrealistic high numbers of midges simulated by Eq. 2. To avoid such artefacts we applied a truncation for the accumulated logarithmic precipitation of 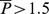 mm/day. This cut-off coincides with annual precipitation maxima of about 1200 mm/year as observed in high Alpine regions [35].

**Figure 6:**
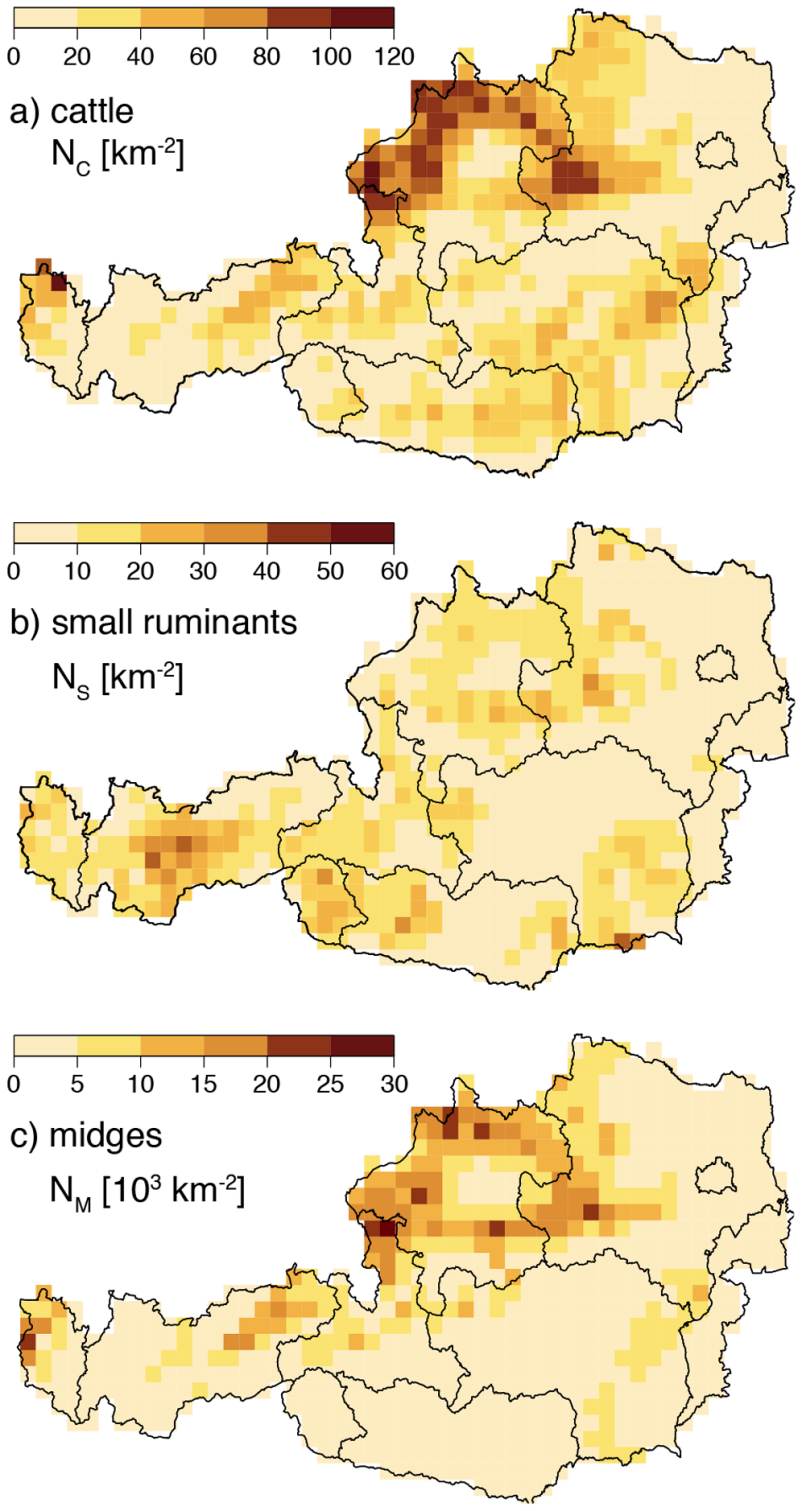
Constant host densities for cattle (a) and small ruminants (b), both given in animals/km 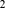, and simulated vector densities for 11 July 2010 in 10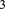 midges/km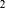 (c).

Host and vector densities used for the 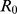 calculation over Austria are depicted in Figure 6. The constant densities of cattle 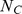 (Figure 6a) and small ruminants 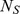 (Figure 6b) were taken from the Austrian veterinary database. The spatial distribution of the vector density 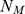, a function of temperature, precipitation, 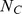 and 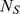, is given in units of 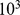 midges/km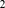. Again we assumed that the simulated midges reflect 1% of the real vector population. Further we adjusted the vector density for host densities following the findings of [36]. Thus, the vector density at a grid point with geographical coordinates 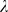 and 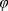 is calculated from the simulated vectors at this coordinate 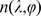 multiplied by a factor of 100 as discussed above and normalized by the host density at the location of the University of Veterinary Medicine Vienna (with 50 ruminants/km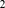).
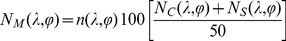
(3)


Note that the vector density varies temporally and spatially, because 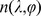 is a function of temperature and precipitation. As an example, Figure 6c depicts the vector density for 11 July 2010.

**Figure 7:**
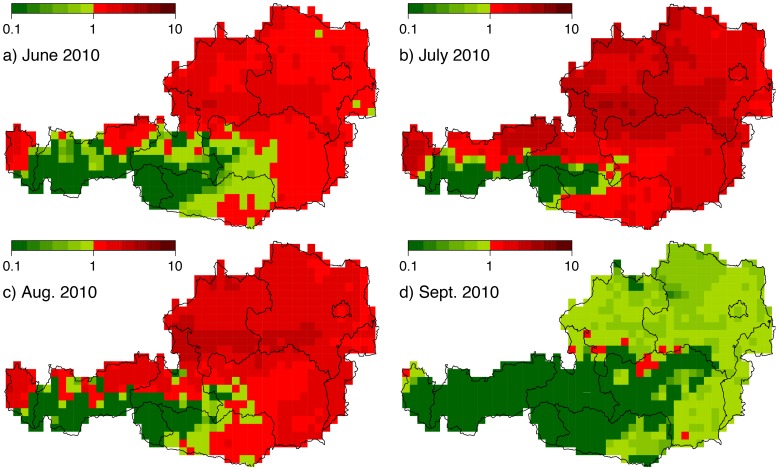
Mean daily basic reproduction number 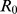 for June to September 2010. The different maps represent the spatio-temporal distribution of the potential risk for a BTV outbreak in Austria. Green colours depict areas not under risk, red under moderate risk and dark red under high risk (note the logarithmic scaling).

Using these daily vector densities, the host densities as well as the parameters and temperature dependent parameter functions we compiled daily risk maps for the period 2010–2011. Movies of simulated daily vector densities and Bluetongue risk maps are provided as supporting information (see Videos S1 and S2) or on our website http://epidemic-modeling.vetmeduni.ac.at/btvmodel.htm. Figure 7 depicts mean daily 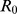 maps for June, July, August, and September 2010. While red areas indicate a potential risk for a major outbreak (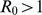), green areas are associated with minor or no risk at all (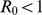). For example, the 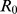 values for July 2010 are within the range 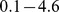, in which the maximum indicates that from one infected animal on average 4.6 animals may be newly infected with BTV. Interestingly areas with a high vector density coincided with high host densities, even without the adjustment after [36]. Verification of the 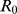 maps is currently not possible, because so far no major BTV outbreaks occurred in Austria. However, the regions with BTV-8 seropositive cases at the Austrian-German border (Figure 1) coincide with regions of high 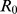 values.

**Figure 8:**
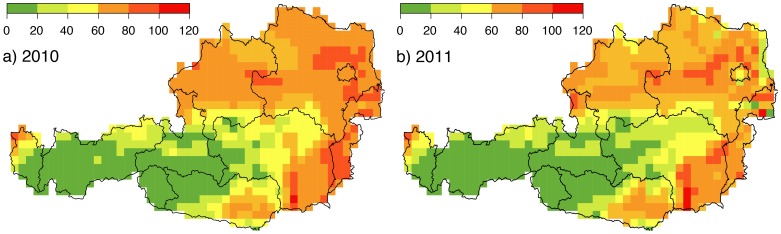
Numbers of days with 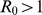 for 2010 (a) and 2011 (b) demonstrating significant differences caused by inter-annual climate fluctuations. Regions with maxima of more than 100 days in the west and north-west of Austria coincide well with confirmed BTV cases depicted in Figure 1.

A visual comparison of the total numbers of days with 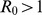 between 2010 (Figure 8a) and 2011 (Figure 8b) shows that in 2010 many regions are at risk for longer, indicating a high inter-annual variation of the 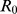 values.

## Discussion

We presented a risk assessment for a potential Bluetongue disease outbreak in Austria depicted by time series and maps of the basic reproduction number 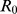. Input data were constant host densities and fluctuating vector densities simulated by a Poisson regression model using temperature and precipitation fields from the Austrian numerical weather prediction model ALADIN. Although the methodology presented here is generally accepted and was recently applied to BTV by [13], [12] and [15], there are some uncertainties in estimating 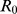.

First of all, the applied 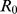 formula is originated from an epidemic model, usually formulated by differential equations, by determining the largest eigenvalue of the so-called next-generation matrix. In the case of BTV it has never been verified by comparison with real outbreak data. Such a verification should demonstrate that the observed BTV-8 dynamics may be approximately reproduced by the underlying epidemic model with the parameter functions selected. Reasons for a missing verification may be due to the fact that it is extremely time-consuming, complex and expensive. However, other epidemic models for arboviral diseases were successfully verified against outbreak data, e.g. for the well-documented Usutu virus outbreak in Vienna [37].

Further, it is difficult to estimate vector densities, even if numbers of trapped midges from a monitoring programme are available. We noticed that the official monitoring data sampled at 54 Austrian locations once a week according to the regulation of the European Union [18] provide an excellent overview on the species composition and on the seasonal activity of our native midge populations. Both were hitherto unknown or at least remain undocumented. A quantitative interpretation of the monitoring data, however, is hardly applicable. This is documented by the following small statistical experiment, where we compiled weekly time series based on Monday catches (after [18]) and compared them with weekly time series based on all daily catches. An explained variance of 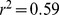 indicates that the numbers of midges sampled according to [18] were afflicted with significant undersampling errors. Further, the official sampling locations were neither representative for their vicinity nor comparable among each other. Also weather stations at short distances to the sampling locations were missing. Therefore, we established our own monitoring with continuously daily observations of midges and environmental parameters from 2009 to present. It allows us to calculate plausible 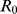 values, although they should be interpreted with care. Due to the generally unknown magnitude of the vector density (we assumed numbers of midges per square kilometre to be two orders of magnitude higher than sampled at a point location), our 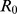 values should be interpreted as relative rather than absolute measures.

Another uncertainty in the vector monitoring lies in the nature of the trapping method. [38] indicated that black light traps are adequate for measuring relative abundance, but not species composition. Further, we don't take into account that midges themselves can be transported by wind over considerable distances, although several models have been developed to investigate this wind-borne spread of BTV [39], [40]. An alternative study on *C. obsoletus* modelling applied a negative binomial regression model (see also Text S1) to simulate the seasonal cycle of daily catches at several locations in England [41]. It distinguishes between the influence of seasonality and meteorological quantities (temperature, precipitation and wind), but does not consider accumulated quantities as defined by CCMs. Further differences comprise the fraction of trapped midges of the *C. obsoletus* complex, which is 86% in Vienna and 48% in England. Unfortunately, due to differences in visualization and verification an objective comparison between [41] and the results presented here is not possible. Both approaches underestimate the extremely high variance of the observations, which is a general problem in vector modelling.

Similar to domestic ruminants most species of wild ruminants, e.g. red deer, are susceptible to BTV infection. We neglected the influence of these potential host species due to their still unclear epidemiological role in BTV transmission [42].

## Supporting Information

Text S1 **Sensitivity study for the extrinsic incubation period and** *C. obsoletus* **models.**
(PDF)

Video S1 **Daily maps of** *C. obsoletus* **densities simulated for May to November 2010 (left) and 2011 (right).**
(MOV)

Video S2 **Daily maps of potential Bluetongue outbreak risk simulated for May to October 2010 (left) and 2011 (right).**
(MOV)
