# Supplementary material for: Bluetongue Disease Risk Assessment Based on Observed and Projected Culicoides obsoletus spp. Vector Densities
Source: PLoS One. 2013 Apr 1;8(4):e60330. doi: 10.1371/journal.pone.0060330 (PMC3613389; doi:10.1371/journal.pone.0060330)
Supplement: Text S1 — Sensitivity study for the extrinsic incubation period and C. obsoletus models. (PDF) [file pone.0060330.s001.pdf]

Text S1

Sensitivity study for the extrinsic incubation period and C. obsoletus models

Katharina Brugger* and Franz Rubel

Institute for Veterinary Public Health, University of Veterinary Medicine Vienna, Veterinärplatz 1, 1210 Vienna, Austria

Sensitivity study for the extrinsic incubation period

In this study the extrinsic incubation period is expressed by its reciprocal, the virus reproduction rate $\gamma_M(T)$. Available functional relationships are depicted in Figure S1. The sensitivity of the basic reproduction number R_0 concerning the application of different temperature dependent functions $\gamma_M(T)$ is depicted in Figure S2. The sensitivity of the Bluetongue risk assessment regarding alternative extrinsic incubation periods is low.

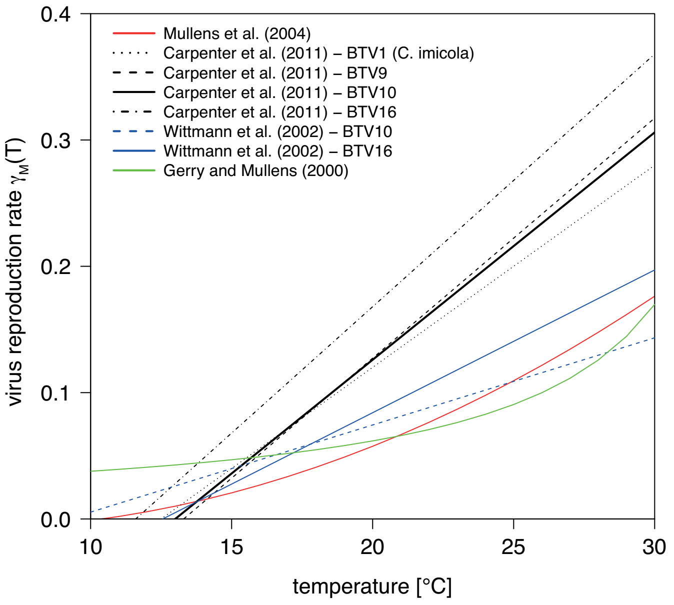

Figure S 1: Temperature dependent virus reproduction rates $\gamma_M(T)$. In red after [1], in black after [2], in blue after [3], and in green after [4].

*Corresponding author. Tel.: +43 1 25077 3533; Fax: +43 1 25077 3590.
E-mail address: katharina.brugger@vetmeduni.ac.at (K. Brugger).

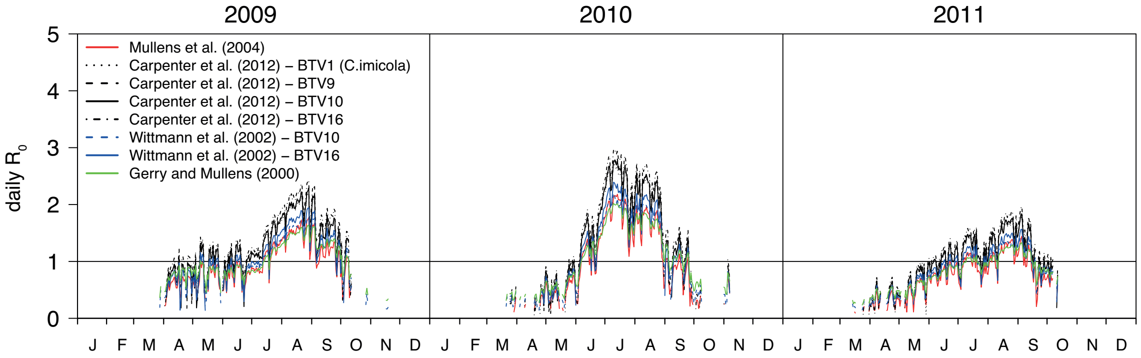

Figure S2: Simulated daily R_0-values for the different virus reproduction rates $\gamma_M(T)$. Location Vienna, period 2009-2011.

Sensitivity study for *C. obsoletus* models

Alternatively, two linear regression models were applied to simulate *C. obsoletus* densities in unsampled regions. The first, a Poisson regression model, gives an accurate estimate of the mean but underestimates the variance. The second, a Negative Binominal regression model, avoids overdispersion but overestimates the mean (Table S1). In this study we prefer the first one. A comparison of Figures 5 and S3 demonstrates the minor impact, i.e. the low sensitivity, of the *C. obsoletus* model on the Bluetongue risk assessment. The verification of the Poisson model results in a sensitivity of 0.81 and a specificity of 0.53. Applying the Negative Binomial model leads to a slightly higher sensitivity of 0.89, but a lower specificity of 0.43.
The formula for the Negative Binomial model reads as follows

$$log_e(n_i + 1) = -1.4991 + 0.0986\ T_{i,i} + 0.1296\ \overline{T}_{i-37,i} + 1.1658\ \overline{P}_{i-100,i-16}\ , \qquad (1)$$

with the daily temperature T, the mean temperature $\overline{T}$, and the mean logarithmic precipitation $\overline{P}$ (all coefficients significant with $p < 0.001$).

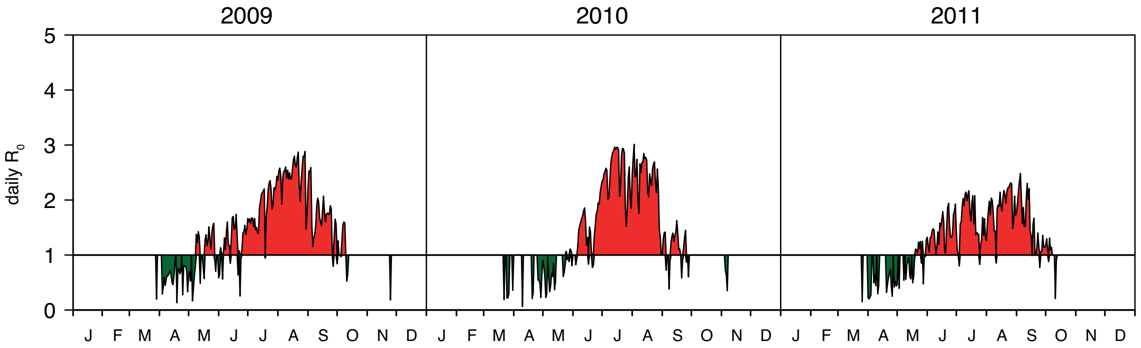

Figure S3: Simulated daily R_0-values based on standard parameters and *C. obsoletus* densities estimated by the Negative Binominal regression model. Location Vienna, period 2009-2011.

	mean	variance
observed midges	14.92	959.91
simulated midges - Poisson regression	14.92	438.88
simulated midges - Negative Binominal regression	19.38	830.17

Table S1: Mean and variance estimated by the Poisson and the Negative Binominal regression model.

References

1. Mullens B, Gerry A, Lysyk T, Schmidtmann E (2004) Environment effects on vector competence and virogenesis of bluetongue virus in culicoides: interpreting laboratory data in a field of context. Veter Ital 40: 160-166.

2. Carpenter S, Wilson A, Barber J, Veronesi E, Mellor P, et al. (2011) Temperature dependence of the extrinsic incubation period of orbiviruses in culicoides biting midges. PLoS ONE 6: e27987.

3. Wittmann EJ, Mello PS, Baylis M (2002) Effect of temperature on the transmission of orbiviruses by the biting midge, Culicoides sonorensis. Med Vet Entomol 16: 147-156.

4. Gerry A, Mullens B (2000) Seasonal abundance and survivorship of *Culicoides sonorensis* (Diptera: Ceratopogonidae) at a sousouth California dairy, with reference to potential bluetongue virus transmission and persistence. J Med Entomol 37: 675-688.
